# Royal Society Discussion Meeting: Utilising the Genome Sequence of Parasitic Protozoa

**DOI:** 10.1002/cfg.88

**Published:** 2001-08

**Authors:** Neil Hall

**Affiliations:** The Sanger Centre Welcome Trust Genome Campus Hinxton, Cambridge CB10 1SA UK

## Abstract

Protozoan parasites cause some of the world’s most important diseases. Genome sequencing information is rapidly being acquired and combined with new developments in functional genome analysis to transform our understanding of parasites, and to enable new approaches to combating the diseases they cause.


**Comparative and Functional Genomics**
*Comp Funct Genom* 2001; **2**: 257–262.
**DOI:** 10.1002/cfg.88

**Meeting Review**

### Royal Society Discussion Meeting: Utilising the genome sequence of parasitic protozoa

### 21-22 March 2001 The Royal Society, 6 Carlton House Terrace, London SW1Y 5AG

Neil Hall*
*The Sanger Centre, Welcome Trust Genome Campus, Hinxton, Cambridge CB10 1SA, UK*

*Correspondence to:
The Sanger Centre, Welcome Trust Genome Campus, Hinxton, Cambridge CB10 1SA, UK.
E-mail: nh1@sanger.ac.uk

### Abstract

**Protozoan parasites cause some of the world's most important diseases. Genome sequencing information is rapidly being acquired and combined with new developments in functional genome analysis to transform our understanding of parasites, and to enable new approaches to combating the diseases they cause. Copyright © 2001 John Wiley & Sons, Ltd.**

**Keywords: parasite; functional genomics; protozoa; apicomplexa; *plasmodium*; *trypanosoma*; *leishmania***

### Introduction

To facilitate the dissemination of the exciting new data, a public discussion meeting, hosted by **The Royal Society**, was organised by **Professor Jennie Blackwell, Professor Chris Newbold, Dr Mike Turner and Professor Keith Vickerman FRS**. The aim was to provide an opportunity for researchers working on many different parasites to discuss and co-ordinate their functional genomics approaches in the light of the wealth of sequence data emerging from the genome centres. Since the *Plasmodium falciparum* (malaria) genome project was initiated in 1996, a broad range of other parasite genomes are now being sequenced, resulting in a sea change in the way that parasite biology can be investigated. The discussion meeting was spilt into four half-day sessions; this report covers a cross-section of the papers presented to an audience of over 270 delegates. A full list of presentations is provided in Table 1.

### Presentations

To provide an example framework for how sequence data can be effectively utilised for functional genomics approaches to cell biology, **Steve Oliver (Manchester, UK)** reported on the experience of the yeast community, who obtained their complete genome sequence back in 1995. In his paper **'Functional genomics: lessons from yeast'** he suggested one of the lessons for the parasite community is that much can be achieved when the community works together, whether this was in the area of genome, transcriptome, proteome, or metabolome. The yeast community saw global expression analysis and high-throughput gene knockouts as a pre-competitive resource. As the annotated yeast genome sequence preceded the development and application of DNA microarray technologies, the community had collaborated in high-throughput northern analysis on all of the yeast genes; genome-wide knockouts were also done as part of a community-wide collaborative endeavour. Laboratories acting independently could never have contemplated this work. It was encouraging that this model of 'community research' had been adopted for the parasite sequencing projects, and was now being applied more and more to protozoan functional genomics approaches. This has been considerably encouraged by the funding agencies, whose policies are increasingly emphasising the importance of multidisciplinary/centre collaborations to answer biological questions.

**Jennie Blackwell's (Cambridge, UK)** presentation **'From genomes to vaccines – *Leishmania* as a model'**

Copyright © 2001 John Wiley & Sons, Ltd.

**Table 1. Presentations**

| | | |
|---|---|---|
| Prof. Jennie Blackwell | Dept.of Medicine, University of Cambridge, UK | From genomes to vaccines: *Leishmania* as a model |
| Dr. Daniel Carucci | Naval Medical Research Center, USA | Technologies for the study of gene and protein expression in *Plasmodium* |
| Prof. Steve Oliver | School of Biological Sciences, University of Manchester, UK | Functional genomics: lessons from yeast |
| Prof Alan Cowman | Walter and Eliza Hall Institute of Medical Research, Melbourne, Australia | Identification and functional analysis of proteins involved in the invasion of human erythrocytes by the malaria parasite *Plasmodium falciparum* |
| Prof. David Roos | University of Pennsylvania, USA | Mining the *Plasmodium* genome database to define organellar functions |
| Prof. Steve Beverley | Washington University, St Louis, USA | Analysis of *Leishmania* gene expression by transposon trapping and expression profiling |
| Prof. Andy Waters | Leiden University Medical Centre, The Netherlands | Demonstration of the orthologous nature of the genomes of *Plasmodium berghei* and *Plasmodium falciparum*: exploitation to understand parasite interactions with both its host and vector |
| Prof. Elisabetta Ullu | University of Yale, USA | Genetic interference by double-stranded RNA in *Trypanosoma brucei* |
| Prof. Ken Stuart | Seattle Biomedical Research Institute and University of Washington, USA | The editing complex of *Trypanosoma brucei* |
| Dr David Sibley | Washington University, USA | Genetic analysis of virulence in toxoplasmosis |
| Prof. Andy Tait | Wellcome Centre of Molecular Parasitology, University of Glasgow | Genetic analysis of phenotype in *Trypanosoma brucei*: a classical approach to potentially complex traits |
| Prof. Alan Fairlamb | University of Dundee | Metabolic analyses in trypanosomes and malaria |
| Prof Richard Moxon | Institute of Molecular Medicine, University of Oxford | Functional genomics of pathogenic bacteria |

Copyright © 2001 John Wiley & Sons, Ltd.

demonstrated the power and pitfalls of DNA microarray technology as a means of assessing gene function. The aim of the work is to identify genes that are expressed in the metacyclic promastigote and amastigote forms of the parasite. It is the metacyclic stage promastigote that invades and survives as an amastigote form in the mammalian host; hence genes expressed at these stages of the life cycle provide possible targets for vaccines. Fortunately, the majority of the life-cycle stages of *Leishmania major* can be mimicked *in vitro*, and RNA profiles can thus be obtained for a wide range of defined developmental points of the life cycle. Initial profiling studies (using EST-based datasets) have been conducted with arrays comprising approximately 1000 of the estimated 8000 *Leishmania* genes; as the proportion of the genome sequenced increases, this work will be extended to all predicted genes of *Leishmania major*. It was clear from the analyses performed so far that clustering of expression profiles (using the EPCLUST software package developed by **Jaak Vilo at the European Bioinformatics Institute**) could identify several genes whose expression characteristics were worthy of confirmation by Northern analysis and further study *in vitro* and *in vivo*.

One observation, also noted by a number of researchers including **Steve Oliver**, working on yeast, and **Steve Beverley**, who reported on his genome survey sequence (GSS) microarray studies in *Leishmania*, was that small conceptual or practical errors in performing microarray experiments frequently lead to misleading results. All stressed the paramount importance of standardisation of assay conditions, particularly if data obtained from different laboratories/experiments were ever to be compared.

**Blackwell's group** are also piloting the use of DNA vaccines in mice as a possible high-throughput tool for identifying new vaccine targets. Initially, this was performed using cDNAs that are thought to be either predominantly, or exclusively, expressed in the amastigote stage of the parasite. Initial experiments were conducted using a pooling strategy, but when single DNA samples from potentially 'protective' pools were used, different results were obtained; DNA vaccines are now being screened individually.

**Dan Carucci (Naval Medical Research Center, USA)** spoke on '**Technologies for the study of gene and protein expression in *Plasmodium***'. He introduced some of the new techniques that are available to analyse the genomes, transcriptomes and proteomes of parasites. These technologies are being utilised by **The Naval Medical Research Center** to develop potential vaccines for the malaria parasite *Plasmodium falciparum*.

The Carucci lab has now made microarray DNA chips for chromosomes 2, 3, 12 and 14 of *P. falciparum* and they plan to extend this to the entire malaria genome over the next two years. These microarrays can be used to study effects of drugs on parasite growth, mechanisms of drug resistance, mechanisms of antigenic variation and genes involved in cell invasion. However, Carucci stressed that for these experiments to be meaningful, numerous replicate experiments must be carried out and that changes in expression levels need to be shown to be statistically significant.

While microarrays can give a good indication of gene expression profiles within the cell, this does not necessarily provide a measure of protein dynamics. The Carucci lab is using high-throughput proteomics to study the protein content of parasite cells. The traditional workhorse of proteomics, the 2D gel, is cumbersome, can be difficult to reproduce reliably, and only displays soluble proteins; hence Carucci has adopted a new technique, capillary liquid chromatography coupled with tandem mass spectrometry, as an alternative. This combined approach facilitates high-throughput analysis of the protein content of a cell or tissue, without the possibly limiting step of electrophoresis.

Carucci also made the point that any laboratory wishing to get heavily involved in proteomics or microarray analysis will need specialised bioinformatics tools to analyse the data efficiently. His laboratory uses an in-house relational database to link information from the malaria genome, microarray experiments and proteomics experiments.

**David Roos, (Pennsylvania, USA)** in his paper **'Mining the *Plasmodium* genome database to define organellar function'** discussed how *Toxoplasma gondii* can be used as a useful model for molecular studies of other *Apicomplexan* parasites. Studies in *Toxoplasma* have elucidated the function of the apicoplast, which up until recently had remained a mystery.

The apicoplast is an organelle, unique to apicomplexan parasites, which is associated with the apical complex of the cell. It has such significant similarity to a plant chloroplast that it is believed to have arisen from a secondary endosymbiotic event of an ancient ancestor of modern plants. The apicoplast

Copyright © 2001 John Wiley & Sons, Ltd.

has a 35 kb genome that encodes mostly housekeeping genes, yet it has since been validated as a prophylactic drug target. By data mining the *P. falciparum* genome sequence, Roos and his colleagues were able to identify a number of genes that were predicted to be localised in the apicoplast; this was achieved by identifying genes exhibiting similarity to chloroplast-encoded genes in plants, or displayed other "plant-like" characterisics. The Roos group were then able to test if the protein products from these, and other genes targeted to the apicoplast are by using a range of GFP fusion constructs in *Toxoplasma*. To date 150 nuclear-encoded plastid genes have been identified, giving an almost complete picture of plastid metabolism.

This work has demonstrated how well designed bioinformatic studies, validated by "wet lab" experimentation, can be a powerful and efficient approach to functional studies. To facilitate this, the Roos lab has established a web-accessible Plasmodium genome database [6], PlasmoDB (**www.plasmodb.org**).

The phenomenon of RNA interference (RNAi) has provided a very useful tool for functional studies of diploid organisms, such as *Drosophila* [5] and *C. elegans* [4]. In her presentation **'Genetic interference by double-stranded RNA in *Trypanosoma brucei*', Elisabetta Ullu (Yale, USA)** demonstrated how it has also been put to use in the *T. brucei* parasite. RNAi uses double-stranded RNA molecules to down-regulate levels of mRNA in cells. This technique has a number of advantages over traditional knockouts, as it avoids the need to transfect organisms twice to knock out both copies of a gene. Also, mutant phenotypes can be rescued using regulated promoters to mediate the degree of interference.

The mechanisms behind gene regulation by RNAi are not well understood; however Ullu and her colleagues have come a long way towards elucidating them. They have identified siRNA (small interfering RNA) species in *Trypanosomes* that are believed to interact with polyribosomes, thus preventing translation. A high proportion of these siRNAs are derived from the retrotransposon-like repeat sequences INGI and SLACS, suggesting that RNAi may play a role in the regulation of these elements.

The RNAi data for *T. brucei* are encouraging, but questions remain. Why, for example, is RNAi proving so hard to demonstrate in the related kinetoplastid *Leishmania*? Time will no doubt tell, but irrespective of this, the observation of RNAi in parasites underlines that they are not only of interest on account of their medical importance, but can frequently provide useful experimental model systems for investigating fundamental biological phenomena. This point was also exemplified by **Ken Stuart** in his presentation entitled **'The editing complex of *Trypanosoma brucei*'**.

In his presentation **'Genetic analysis of phenotype in *T. brucei*: a classical approach to potentially complex traits'**, **Andy Tait (Glasgow, UK)**, spoke about the relevance of genetic studies in the post-genomic era. While genetic analysis is typically not a high throughput technique for functional analyses, it has its advantages in that the work is biologically driven with a defined phenotype that usually can be chosen for its scientific relevance, such as drug resistance or reduced infectivity; complex phenotypes are notoriously difficult to study by gene knockouts or RNAi.

Tait and his colleagues now have a genetic map of *Trypanosoma brucei* mini-satellite and micro-satellite markers at a resolution of 10 cM on chromosomes I and II, and a partial map of chromosome IV. They also have 140 AFLP markers, giving a total map covering 1/3 of the genome.

The Tait group intends to complete this map over the next few years. Stocks of *T. brucei* have been identified that differ in their drug sensitivity, human infectivity and virulence, and these parental stocks are being crossed to build up a series of progeny panels. Linkage analysis is to be used to determine the genetic bases underlying these important phenotypes.

In his presentation **'Demonstration of the orthologous nature of the genomes of *Plasmodium berghei* and *Plasmodium falciparum*: exploitation to understand parasite interactions with both its host and vector' Andrew Waters (Leiden, The Netherlands)** spoke about how comparative genomics is starting to aid our understanding of the structure and content of malaria genomes. The genomes of *Plasmodium spp* have been shown to be highly syntenic [2,1]. Synteny is a measure of genetic conservation, which may refer to entire chromosomes or simply neighbouring segments of DNA. It does not, as is commonly thought, simply measure gene order.

Prior to genome sequencing, synteny was measured by hybridising DNA probes to pulsed field gel electrophoresis (PFGE)- separated chromosomes. It

Copyright © 2001 John Wiley & Sons, Ltd.

was observed that rodent malarias were highly syntenic, with almost all probes hybridising to equivalent chromosomes in all species analysed. There is also significant synteny between rodent malarias and the human parasite, *P. falciparum.*

Waters demonstrated how synteny on a smaller scale (i.e. conservation over small genetic distances) can aid the annotation and analysis of genome sequence data.

Waters and his collaborators have fully sequenced a YAC clone containing DNA from the rodent malaria species *Plasmodium berghei.* The sequence obtained from this clone has been carefully analysed and the primary transcipt RNA splicing characteristics of the 6 predicted genes annotated. The *P. berghei* YAC is highly syntenic with a contig sequenced and assembled by **The Institute of Genomic Research (TIGR, Washington)** from chromosome 10 of *P. falciparum.* When the two sequences are compared, it is clear that the coding regions are considerably more conserved than non-coding regions, hence it is possible to directly compare the predicted genes and splicing patterns between the two species.

Importantly, this work demonstrates that given a well-studied sequence from one *Plasmodium* species, conserved DNA synteny will enable us to identify structural components and coding regions in other species. Variations from this 'rule' will serve as flags for areas containing potentially interesting biological features (e.g. host restriction). Also, even two syntenic, but otherwise uncharacterised sequences could give clues to the location and structure of genes within both of them, due to the inherent conservation between the coding regions.

In his presentation '**Metabolic analysis in trypanosomes and malaria**', **Alan Fairlamb (Dundee, UK)** concentrated on the analysis of metabolic pathways in parasitic organisms. Such analysis is critical for the identification of potential drug targets. In theory, the availability of a completely annotated genome sequence should provide investigators with a complete metabolome that should in turn provide a wide array of possible drug targets. However, Fairlamb went on to explain that the analysis of such a complex data set requires well-designed and well-curated metabolism databases.

As an example, Fairlamb discussed the polyamine biosynthesis pathway in the kinetoplastids *Leishmania* and *Trypanosoma*; in these protozoan parasites, the enzyme trypanothione reductase is present, "replacing" the glutathione reductase found in the mammalian pathway [3].

This difference results in a unique metabolite, trypanothione (N1, N8-bis(glutathionyl)-spermidine), which has taken on many of the protective and anti-oxidant functions normally ascribed to glutathione in mammalian cells. Inhibitor studies have validated the parasite pathway as a possible drug target. However in some public metabolic pathway databases, this novel pathway is not represented, despite the breadth of knowledge available.

This is an unsatisfactory state of affairs, as components of pathways that are unique to, or different in, medically relevant organisms are more than likely to be candidate drug targets. Intensified dialogue between the research communities, and those curating the public databases, is a priority for all scientists involved in the field of parasite functional genomics.

**Chris Newbold (Oxford, UK)** closed by thanking everyone for their contribution to the discussions, and the **Royal Society** for hosting the meeting. In keeping with the number of different organisms that were the subject of the talks over the two days, he suggested that perhaps one of the most important messages from the meeting was that a great deal of information would come from comparative genomics. With so many genomes being sequenced now by centres around the world, parasitic protozoa offer an exciting opportunity to understand genome evolution, possibly more so than in any other area of eukaryotic biology. It was also very important that the communities take on board the concept of pre-competitive research, particularly if the huge quantity of sequencing, expression and proteomics data, to cite but three examples, were to be used to their full potential.

### References

1. Carlton JM, Galinski MR, Barnwell JW, Dame JB. Karyotype and synteny among the chromosomes of all four species of human malaria parasite. *Mol Biochem Parasitol* 1999; **101**: 23–32.
2. Carlton JM, Vinkenoog R, Waters AP, Walliker D. Gene synteny in species of *Plasmodium. Mol Biochem Parasitol* 1998; **93**: 285–294.
3. Fairlamb AH, Cerami A. Metabolism and functions of trypanothione in the *Kinetoplastida. Annu Rev Microbiol* 1992; **46**: 695–729.
4. Fire A, Xu S, Montgomery MK, Kostas SA, Driver SE, Mello CC.*Potent and specific genetic interference by*

Copyright © 2001 John Wiley & Sons, Ltd. 

*double-stranded RNA in Caenorhabditis elegans.: Nature* 1998; **391**: 806–811.

5. Hammond SM, Bernstein E, Beach D, Hannon GJ. An RNA-directed nuclease mediates post-transcriptional gene silencing in *Drosophila* cells. *Nature* 2000; **404**: 293–296.
6. The Plasmodium Genome Database Collaborative. PlasmoDB: An integrative database of the *Plasmodium falciparum* genome. Tools for accessing and analyzing finished and unfinished sequence data. *Nucleic Acids Res* 2001; **29**: 66–69.

---

The Meeting Reviews of *Comparative and Functional Genomics* aim to present a commentary on the topical issues in genomics studies presented at a conference. These reviews are invited and each represents a personal critical analysis of the current reports and aim at providing implications for future genomics studies.

Copyright © 2001 John Wiley & Sons, Ltd.

## References

[PDF_00407] Carlton J. M., Galinski M. R., Barnwell J. W., Dame J. B. (1999). Karyotype and synteny among the chromosomes of all four species of human malaria parasite.. Mol Biochem Parasitol.

[PDF_00411] Carlton J. M., Vinkenoog R., Waters A. P., Walliker D. (1998). Gene synteny in species of Plasmodium.. Mol Biochem Parasitol.

[PDF_00414] Fairlamb A. H., Cerami A. (1992). Metabolism and functions of trypanothione in the Kinetoplastida.. Annu Rev Microbiol.

[PDF_00423] Hammond S. M., Bernstein E., Beach D., Hannon G. J. (2000). An RNA-directed nuclease mediates post-transcriptional gene silencing in Drosophila cells.. Nature.

